# The role of serum creatine kinase levels in anterior cervical spinal surgery: Change trends and risk factors

**DOI:** 10.1097/MD.0000000000028300

**Published:** 2021-12-23

**Authors:** Peiming Sang, Yanyan Ma, Binhui Chen, Ming Zhang

**Affiliations:** a Department of Orthopedic Surgery, Lihuili Hospital of Ningbo Medical Center, Ningbo, Zhejiang, PR China; b Department of Gastroenterology, Lihuili Hospital of Ningbo Medical Center, Ningbo, Zhejiang, PR China.

**Keywords:** anterior cervical spinal surgery, creatine kinase, iatrogenic invasiveness, risk factors

## Abstract

This study aimed to describe change trends in serum creatine kinase (CK) values for patients undergoing anterior cervical spinal surgery and identify risk factors that affect the CK values perioperatively, intending to decrease the degree of the iatrogenic invasiveness of the procedure.

We retrospectively analyzed 122 patients undergoing anterior cervical spinal surgery from January 2019 to May 2020. For all patients, CK level was measured 1 day before the operation. Daily CK levels were evaluated on postoperative days 1 to 7. These data were analyzed in terms of age, gender, the use of microendoscopy during surgery, the number of cervical operative segments, and operative methods to determine whether these risk factors influenced postoperative CK increases.

A total of 122 patients were enrolled. The preoperative average CK level was 72.7 U/L, and the average CK levels were 130.6, 122.4, 99.1, 82.8, 73.7, 63.9, and 55.4 U/L from the postoperative day (POD) 1 to POD7, respectively. CK level changes on POD1 increased with the number of operated cervical segments. However, changes were not related to age, gender, microendoscopy, or the operative method.

Increased serum CK level was associated with the number of cervical operative segments, rather than age, gender, the use of microendoscopy, or the operative methods. These findings suggest that the number of cervical operative segments determined the degree of iatrogenic injury for anterior cervical spinal surgery.

## 1 Introduction

The anterior cervical muscle group, the airway, esophagus, and prevertebral soft tissue are pulled into the contralateral position during anterior cervical spinal surgery, leading to dysphagia and dyspnea. Increased intraoperative invasiveness correlates with complications; therefore, it is critical to identify and avoid risk factors for iatrogenic injury during anterior cervical spinal surgery.

Postoperative serum creatine kinase (CK) levels are parameters for assessing muscle injury from various spinal procedures^[1]^ and evaluating degrees of iatrogenic injury. CK level significantly correlates with the length and depth of the surgical dissection,^[2]^ and a significant relationship was found between CK levels and duration and intensity of the pressure on paraspinal muscles exerted by retraction.^[1,3,4]^

To date, no studies have focused on changes in CK level or risk factors for elevated CK levels after anterior cervical spinal surgery. Therefore, in the present study, we aimed to define the range of CK values and the factors affecting those values to reduce the levels of muscle injury by reducing the causative factors and avoiding iatrogenic injury during surgery.

## 2 Materials and methods

### 2.1 Study design

The institutional review board approved the terms of the present study (No.KY2019PJ057), and informed consent was obtained from all patients. This study was performed using patients undergoing anterior cervical spinal surgery from January 2019 to May 2020.

All patients with anterior cervical spinal surgery were considered for participation. The inclusion criteria included age ≥18 years, primary anterior cervical spinal surgery, including anterior cervical discectomy fusion (ACDF) and anterior cervical corpectomy fusion (ACCF). Exclusion criteria included the presence of traumatic injuries, surgical intervention for underlying infection, tumor or pregnancy, muscle injury or eccentric exercise, or a personal history of rhabdomyolysis,^[5]^ all of which could affect CK level before surgery.

At the standard procedure, CK level was measured 1 day before surgery for all patients, and daily CK levels were measured at 1 to 7 days after surgery. The operative mode was recorded (i.e., number of segments, operative methods).

### 2.2 Statistical analysis

Statistical analyses were performed using SPSS ver.16.0 (SPSS Inc., Chicago, IL). The normality of the data was evaluated. If they were non-normally distributed, the Student *t* test was assessed to compare the results between 2 groups. If they were normally distributed, the independent samples *t* test was used to compare its. One-way analysis of variance and least-squares difference *t*-tests were used to compare the outcomes among the 3 groups. A correlation analysis with Pearson's correlation coefficient was performed to test the relationship between preoperative CK value, postoperative day (POD) 1 CK value, CK change at POD1, and age. The level of significance was set at *P* < .05.

## 3 Results

A total of 122 patients underwent anterior cervical spinal surgery, and all were followed for 1 week after surgery with daily CK measurements (Table 1). The distribution of data was normal (*P* > .05). CK values increased significantly after surgery, peaking at POD1 and decreasing to normal by POD4. The difference in preoperative and POD1 CK levels represented the invasiveness of the surgery.

**Table 1:** Variables and average CK levels.

Variable	Number of patients	Mean ± standard deviation	Median	Min	Max
Preoperative CK (U/L)	112	72.7 ± 26	79	18	130
POD1 CK(U/L)	112	130.6 ± 48.1	124	57	286
POD2 CK(U/L)	112	122.4 ± 68.7	106.5	51	360
POD3 CK(U/L)	112	99.1 ± 54.5	83.5	40	274
POD4 CK(U/L)	112	82.8 ± 53.9	63	29	343
POD5 CK(U/L)	112	73.7 ± 48.5	55	30	292
POD6 CK(U/L)	112	63.9 ± 39.8	50	26	200
POD7 CK(U/L)	112	55.4 ± 34.1	45	20	186
CK change at POD1 (U/L)	112	57.9 ± 44.4	49.5	−40	191

CK = creatine kinase, POD = postoperative day.

We determined whether various risk factors (age, gender, the use of microendoscopy during surgery, the number of operative segments, and operative methods) affected CK level (Table 2). We found that CK levels were not related to age (*P* > .05).

**Table 2:** Correlation of CK level with the age.

Age	Variable	Mean ± standard deviation	Median	Min	Max	Pearson correlation (r-value)	*P* value
58.96 ± 10.08	Preoperative CK	72.7 ± 26	79	18	130	−0.125	.1907
58.96 ± 10.08	CK value at POD1	130.6 ± 48.1	124	57	286	−0.012	.8995
58.96 ± 10.08	CK change at POD1	57.9 ± 44.4	49.5	−40	191	0.06	.5306

CK = creatine kinase, POD = postoperative day.

The effects of gender on CK value were shown in Table 3. CK values were higher for men than women preoperatively and at POD1; however, there was no significant difference between men and women for CK change at POD1, suggesting that gender did not affect CK change perioperatively (*P* > .05).

**Table 3:** CK value as a function of patient gender.

Variable	Gender	Number of patients	Mean ± standard deviation	Median	Min	Max	Statistic value	*P* value
Preoperative CK	Male	64	80.1 ± 24.9	87.5	29	130	3.668	3.787 × 10^–4^
	Female	48	62.9 ± 24.3	65.5	18	100		
CK value at POD1	Male	64	144.97 ± 50.9	138.5	69	286	3.86	1.911 × 10^–4^
	Female	48	111.5 ± 36.5	102	57	213		
CK change at POD1	Male	64	64.8 ± 45.4	51.5	10	191	1.929	.0563
	Female	48	48.7 ± 41.8	49	−40	171		

CK = creatine kinase, POD = postoperative day.

The effect of surgery with microendoscopy was shown in Table 4. There were no significant differences in CK levels between surgery with microendoscopy and traditional surgery groups preoperatively, at POD1, and CK change (*P* > .05).

**Table 4:** Comparison of the CK levels for surgery with microendoscopy or not.

Variable	Surgery method	Number of patients	Mean ± standard deviation	Median	Min	Max	Statistic	*P* value
Preoperative CK	With microendoscopy	22	68.68 ± 23.49	73	36	120	0.812	.419
	Without microendoscopy	90	73.7 ± 26.6	80	18	130		
CK value at POD1	With microendoscopy	22	134.8 ± 60.09	118	73	286	−0.453	.652
	Without microendoscopy	90	129.6 ± 45	124	57	242		
CK change at POD1	With microendoscopy	22	66.1 ± 4.94	56	−10	191	−0.968	.335
	Without microendoscopy	90	55.9 ± 4.32	49	−40	171		

CK = creatine kinase, POD = postoperative day.

In terms of the effect of operative methods and the number of cervical segments operated, the patients were divided into 5 groups according to the number of levels: 1 segment of ACDF (n = 35), 2 segments of ACDF (n = 26), 3 segments of ACDF (n = 8), 1 segment of ACCF (n = 23), and 1 segment of ACCF with 1 segment of ACDF (n = 20). The preoperative CK, POD1 CK, and CK change at POD1 are shown in Table 5.

**Table 5:** The CK levels of these groups.

Variable	Group	Number of patients	Mean ± standard deviation	Median	Min	Max
Preoperative CK	1ACDF	35	69.2 ± 22.17	69	32	112
	2ACDF	26	83.3 ± 21.29	88	36	114
	3ACDF	8	64.38 ± 33.78	58.5	33	120
	1ACCF	23	72.26 ± 23.67	74	29	100
	1ACCF with 1ACDF	20	69.1 ± 34.4	74	18	130
CK value at POD1	1ACDF	35	105.9 ± 36.27	98	57	213
	2ACDF	26	145.3 ± 32.27	141	92	214
	3ACDF	8	172.5 ± 80.8	159.5	85	286
	1ACCF	23	127.1 ± 34.2	124	75	183
	1ACCF with 1ACDF	20	142.2 ± 60.96	131.5	71	242
CK change at POD1	1ACDF	35	36.7 ± 42.95	28	−40	171
	2ACDF	26	62 ± 24.89	57	32	114
	3ACDF	8	108.13 ± 50.44	103	48	191
	1ACCF	23	54.8 ± 42.44	42	13	154
	1ACCF with 1ACDF	20	73.15 ± 47.1	66	9	139

ACCF = anterior cervical corpectomy fusion, ACDF = anterior cervical discectomy fusion, CK = creatine kinase, POD = postoperative day.

The effect of the number of operative segments was shown in Table 6. There were significant differences in CK values at POD1 and CK change at POD1 among different segments of ACDF (*P* < .05).

**Table 6:** Comparison of CK level among different segment of ACDF.

Variable	Group	Number of patients	Mean ± standard deviation	Median	Min	Max	*F* value	*P* value
Preoperative CK	1ACDF	35	69.2 ± 22.17	69	32	112	1.58	.185
	2ACDF	26	83.3 ± 21.29	88	36	114		
	3ACDF	8	64.38 ± 33.78	58.5	33	120		
CK value at POD1	1ACDF	35	105.9 ± 36.27	98	57	213	5.526	4.399 × 10^–4^
	2ACDF	26	145.3 ± 32.27	141	92	214		
	3ACDF	8	172.5 ± 80.8	159.5	85	286		
CK change at POD1	1ACDF	35	36.7 ± 42.95	28	−40	171	6.194	1.601 × 10^–4^
	2ACDF	26	62 ± 24.89	57	32	114		
	3ACDF	8	108.13 ± 50.44	103	48	191		

ACDF = anterior cervical discectomy fusion, CK = creatine kinase, POD = postoperative day.

To assess the effect of operative methods on CK value for the same number of operative segments, it was necessary to compare the differences of CK value between the 2ACDF and the 1ACCF groups and between the 3ACDF and the 1ACCF with the 1ACDF group. The differences between the 2ACDF and 1ACCF groups were shown in Table 7. There was no significant difference in CK levels between them (*P* > .05).

**Table 7:** Comparison of CK levels between 2ACDF group and 1ACCF group.

Variable	Group	Number of patients	Mean ± standard deviation	Median	Min	Max	*t* value	*P* value
Preoperative CK	2ACDF	26	83.3 ± 21.29	88	36	114	−1.72	.092
	1ACCF	23	72.26 ± 23.67	74	29	100		
CK value at POD1	2ACDF	26	145.3 ± 32.27	141	92	214	−1.918	.061
	1ACCF	23	127.1 ± 34.2	124	75	183		
CK change at POD1	2ACDF	26	62 ± 24.89	57	32	114	−0.732	.468
	1ACCF	23	54.8 ± 42.44	42	13	154		

ACCF = anterior cervical corpectomy fusion, ACDF = anterior cervical discectomy fusion, CK = creatine kinase, POD = postoperative day.

The differences between the 3ACDF group and the 1ACCF with 1ACDF group were shown in Table 8. There were no significant differences between them (*P* > .05).

**Table 8:** Comparison of CK levels between 3ACDF group and 1ACCF with 1ACDF group.

Variable	Group	Number of patients	Mean ± standard deviation	Median	Min	Max	*t* value	*P* value
Preoperative CK	3ACDF	8	64.38 ± 33.78	58.5	33	120	0.326	.747
	1ACCF with 1ACDF	20	69.05 ± 34.44	74	18	130		
CK value at POD1	3ACDF	8	172.5 ± 80.8	159.5	85	286	−1.083	.289
	1ACCF with 1ACDF	20	142.2 ± 60.96	131.5	71	242		
CK change at POD1	3ACDF	8	108.13 ± 50.44	103	48	191	−1.742	.093
	1ACCF with 1ACDF	20	73.15 ± 47.06	66	9	139		

ACCF = anterior cervical corpectomy fusion, ACDF = anterior cervical discectomy fusion, CK = creatine kinase, POD = postoperative day.

## 4 Discussion

Iatrogenic muscle injury is inevitable after anterior cervical spinal surgery. Because of the need to increase the space for operating, the anterior cervical muscle group is pulled into the contralateral position, leading to iatrogenic muscle injury. There is a relationship between the degree of surgical invasiveness and the influence of the surgical route on the extent of muscle injury.^[6,7,8,9]^

Biochemical changes resulting from muscle damage are easily measured, with CK value being the most sensitive marker.^[10,11]^ CK is present in many organs and tissues, where it catalyzes the conversion of creatine and adenosine triphosphate to phosphocreatine and vice versa.^[12]^ It has served as a biomarker of muscle damage and iatrogenic invasiveness.^[13]^ In the present study, we found that, compared with preoperative values, CK values after surgery peaked at POD1, decreasing subsequently and returning to normal at POD4, suggesting that iatrogenic swelling of prevertebral soft tissue (including the airway and esophagus) was most pronounced at POD1. On the first postoperative day, it is critical to monitor frequently for obstruction of the airway and swallowing dysfunction. Dyspnea must be regarded as a life-threatening emergency.

There was no significant difference in CK level concerning age in the present study, a finding that differed from other studies. Kang et al found that increasing age correlated with decreased paraspinal musculature as muscle fibers were replaced by fibrous tissue or fatty infiltration, which would cause a reduction in the effective muscle area at the lumbar spine.^[14]^ This finding suggests that CK levels should decrease with the increasing age. There are several reasons for the discrepancy between the present study and Kang, due to the rich for blood, getting better of anterior cervical muscle group attributed to swallowing and cervical lordosis, not the same as the lumbar spine.

Though the CK values differed between the genders at preoperative and POD1, the difference was not significant. However, other studies found that CK elevation after lumbar spine surgery was higher in men than in women, most likely due to differences in muscle mass.^[15–17]^ These results were not like ours, which we attributed to muscle mass differences between lumbar and cervical areas.

Concerning the use of the operating microscope, there were no differences in CK values. Previous studies reported that the operating microscope offers advantages for spinal surgery. Rock et al reported significant improvement in patient satisfaction and hospital stay after using the operating microscope.^[18]^ Damodaran et al found that the advantages of the microscope included better illumination, magnification, and coaxial vision.^[19]^ The microscope could help avoid complications during decompression of spinal cord and nerve roots through better illumination and magnification.^[20]^ However, due to the implantation of the plate, the area of exposure was not reduced using the microscope. Compared to traditional surgery, the differences in CK value were not significant with using a microscope for anterior cervical spinal surgery.

We found that CK values correlated with the number of segments for anterior cervical spinal surgery. Higher numbers of operative segment sentail larger operative fields and consequent higher CK levels. McCarthy et al suggested that multilevel fusions for anterior cervical spinal surgery posed a higher risk of complications than single-level fusion; these complications included pseudoarthrosis, adjacent segment disease, sagittal imbalance, and construct subsidence.^[21]^ In clinical practice, it was critical preoperatively to determine the position where the spinal cord or nerve root is being compressed. Surgery aims to remove the compressed material and eliminate the symptom while decreasing the operating segments to reduce iatrogenic invasiveness and complications.

In the present study, CK values were not associated with methods of surgery containing ACCF and ACDF. ACCF and ACDF have been common in cervical surgery for single-level cervical spondylotic myelopathy and treating multilevel disease. There is some controversy about the choice of ACDF and ACCF for anterior cervical spinal surgery.^[22]^ Oh et al found that surgical management of two-level cervical spondylotic myelopathy using ACDF or ACCF was similar in terms of the clinical outcomes and that two-level ACDF was superior to one-level ACCF in terms of operation times, bleeding amounts, and radiologic results.^[23]^ Banno et al found that ACCF was associated with worse clinical outcomes than ACDF following multilevel treatment for cervical spondylotic myelopathy.^[24]^ In terms of number of operative segments, two-level ACDF was equal to one-level ACCF for treating two-level anterior cervical spinal disease. However, for two-level ACDF, the procedure was performed with one segment by 1 segment, for one-level ACCF, it was achieved with 2 segments simultaneously. Thus, it was difficult to compare the degree of invasiveness between two-level ACDF and one-level ACCF. In the present study where we measured CK preoperatively, two-level ACDF was identical to one-level ACCF concerning the degree of iatrogenic injury.

### 4.1 Limitations

This study has some significant limitations. First, the sample sizes relatively small, the standard deviations of CK values are high, and the follow-up period is relatively short, which may reduce the accuracy. Second, traction forces and time during surgery may affect CK values. Third, we did not include patients undergoing revision surgery, surgery without anterior cervical plate fixation, or surgery with artificial cervical disk replacements.

## 5 Conclusion

CK values should be used to assess the degree of surgical invasiveness for anterior cervical spinal surgery. The change of postoperative CK value is not related to age, gender, using microendoscopy during surgery, or operative methods. However, it is closely associated with the number of cervical operative segments. To reduce iatrogenic injuries, it is recommended to reduce the number of cervical operative segments while ensuring postoperative efficacy. So it is very significant for doctors to identify the segments of cervical spinal disease that are responsible for patients’ symptom preoperatively, which should be treated using ACCF or ACDF. And it is important to avoid treating the segments unrelated to patients’ symptom preoperatively. Only in this way, the degree of surgical invasiveness for anterior cervical spinal surgery can be minimized.

## Author contributions

**Conceptualization:** Binhui Chen.

**Data curation:** Peiming Sang, Binhui Chen, Ming Zhang.

**Formal analysis:** Yanyan Ma.

**Funding acquisition:** Yanyan Ma.

**Investigation:** Yanyan Ma.

**Methodology:** Peiming Sang, Binhui Chen, Ming Zhang.

**Project administration:** Ming Zhang.

**Software:** Peiming Sang, Yanyan Ma, Ming Zhang.

**Supervision:** Peiming Sang, Yanyan Ma, Binhui Chen.

**Validation:** Ming Zhang.

**Visualization:** Peiming Sang, Ming Zhang.

**Writing – original draft:** Peiming Sang.

**Writing – review & editing:** Peiming Sang.
